# Ringworm: Its Nature, Diagnosis, and Treatment

**Published:** 1897-01-09

**Authors:** David Walsh

**Affiliations:** Physician Western Skin Hospital, London; Clinical Assistant Blackfriars Skin Hospital.


### RINGWORM: ITS NATURE, DIAGNOSIS, AND TREATMENT.

By David Walsh, M.D.Edin, Physician Western Skin Hospital, London; Clinical Assistant Blackfriars Skin Hospital.

During recent years the malady known as "ringworm" has attracted a good deal of attention, mainly on account of its prevalence, and of the interesting discoveries that have been made in the natural history of the fungus to which it is due. As to its increase, there appears to be a fairly general agreement of opinion to that effect among both specialists and general practitioners. From time to time we hear of its frequency in Board schools, and it is a fact that most of the children brought to skin hospitals suffering from ringworm of the scalp are attendants at institutions of that kind. Moreover, such patients often state that a great many of their fellow-scholars are affected in a similar manner.

Board schools, apparently on good grounds, have been credited with no small share in the recent increase of diphtheria throughout the country. The truth of that explanation, however, has been denied in some quarters, but the most ardent upholders of the present school system would hardly assert that it has not much to answer for in the spread of ringworm. The facts of the case are simple. In ringworm of the scalp we have a highly contagious complaint, and in the schools we bring together large numbers of children, who constitute, as it were, an open field for the growth of the invading fungus. The same may be said of Poor Law schools, where ringworm is often extremely common, despite the care with which it is usually treated.

Unfortunately, ringworm of the scalp is regarded by many persons as a slight affection, that calls for little or no treatment. So far from that being the case, however, it is from many points of view a serious malady. For instance, in spite of skilled and careful treatment, it may last for months or even years. It is apt to give rise to various secondary inflammatory troubles. It may leave behind in the scalp a lifelong seborrhœa, that condition of mild chronic inflammation which is now recognised as a fruitful source of greyness, thin hair, baldness, and as the starting-point of not a few skin eruptions. Lastly, each child whom it attacks becomes a centre of further infection. For these and other reasons ringworm of the scalp is often a most troublesome malady, costly, tedious, and difficult to cure, entailing present suffering and future disfigurement upon the individual. Its ravages lay such a tax upon the community, directly and indirectly, that the matter must sooner or later be forced upon the serious attention of the Education Department. For some time past there have been indications of a tendency on the part of the Local Government Board to treat ringworm in Poor Law schools as they now deal with ophthalmia, namely, by strict isolation and skilled special treatment. These desirable ends are attained by an "isolation school," where the education of inmates is kept up. The plan works admirably in regard to ophthalmia, and might well be extended to the widespread and disastrous scourge of ringworm.

**Recent Discoveries as to the Essential Fungus.**—The discovery of the trichophyton was announced by Gruby in 1841. The class of fungus to which it belongs is still uncertain. Declaux and Leslie Roberts cultivated the trichophyton fungus outside the body, and the latter observer reproduced the original disease by inoculation. Sabouraud, however, has made great advances in our knowledge of the subject within the last few years. He showed that the two chief varieties in man were a large-spored (megalo-sporon), and a small-spored (micro-sporon), the two never being found together on the same head. Without going into minute details, his broad conclusions may be stated as follows:—

1. The small-spored tinea fungus (Micro-sporon Audouini) is by far the more common in children, being met with in two-thirds of the cases. Its mycelium tubes grow in the substance of the hair, but the spores form a dense "mosaic" layer or sheath round its shaft. There are few species of the micro-sporon, and, with the exception of one found upon the horse, they are human parasites. It is the common fungus of scalp ringworm, and is rebellious to treatment.

2. The large-spored variety has many species, and is contracted by man from various lower animals, as the dog, horse, or cat. It is essentially the fungus of ringworm of the beard and of the smooth parts of the skin. As a general rule it is much more readily overcome by treatment than the small-spored forms.

These researches, which proved the plurality of the ringworm fungi, have been extended by many observers. In England, Adamson (*Journal of Dermat.*, July, 1895) has published a capital account of his work in that direction, which in the main confirms the conclusions of Sabouraud. He finds, however, that the small-spored variety is by far the more common in England, for it occurred in 173 out of 178 scalp cases, the remaining five being large-spored. In a later account he stated the proportion to be 95 per cent. in 263 consecutive cases. When cultivated outside the body in an artificial medium (Sabouraud uses a peptone-maltose agar-agar) the micro-sporon shows a downy surface and white colour, whereas the megalo-sporon has a powdery surface with branching rays and often a yellowish tint.

Notwithstanding these recent additions to our knowledge, no great advances have been made in treatment. The main difficulty in the treatment of ringworm is a mechanical one, the fungus burrowing into the substance of the hair, as well as into the hair follicles and deeper layers of the epidermis, where it is beyond the reach of parasiticide remedies. A large-spored patch on skin where the hair is rudimentary (lanugo), as at the back of the hand, may often be cured with a single application of tincture of iodine. In the same way a megalo-sporon patch on the scalp may be sometimes rapidly cured. But take a chronic ring on the head, where the hair has broken away, leaving only a scanty stubble of dark stumps, and one may paint on iodine day after day for a month with little effect. In the last-mentioned case the fungus has got into the roots of the hair and the deeper layers of the surface-skin. In fact, to cure a chronic ringworm requires a large amount of resource, together with the utmost patience and thoroughness of treatment. Considering the infectiveness of the fungus, and the ease with which its myriads of spores may be spread about, the wonder is that this pest of mankind is not far more universal.

**Methods of Infection.**—Among school children the most common mode of infection is by the accidenta or playful exchange of caps. It may be mentioned, in passing, that a cap is not wanted out of doors by the average healthy child in mild weather. Brushes and combs, pillows and bedclothes, and, to a less extent, towels, no doubt often convey the disease. Now and then domestic animals, as cats, dogs, or horses, are found to be answerable. In a great many cases, however, it is impossible to trace the origin of the mischief.

**Reinfection and Multiple Infection.**—A patient suffering from ringworm of the head runs the risks of reinfection no less than of multiple infection from his head-gear. The lining of his hat or cap becomes laden with spores, which may either start the disease again when cured or plant it in fresh parts of the scalp. It is extremely common for children affected with ringworm to wear long worsted "fisher" caps. Nothing could be worse from a medical point of view. The material gets loaded up with short hair stumps and spores from the diseased patches—as may be readily proved by the microscrope—so that in pulling the tightly-fitting cap into position the seeds of infection are scattered broadcast over the whole scalp. At any rate, some of the worst cases that have come under the writer's observation have occurred in children wearing these "fisher" caps. The way to avoid the danger is simple, and will be described under the heading of prevention.

